# Climate policy and the concept of co-benefits in India

**DOI:** 10.1007/s40847-023-00235-2

**Published:** 2023-02-28

**Authors:** Theresa Stahlke

**Affiliations:** Department of Economics, TU Bergakademie Freiberg, 09596 Freiberg, Germany

**Keywords:** Climate policy, Co-benefits, Developing country, Differentiated responsibilities, India, Mitigation of climate change, NAPCC, Paris agreement, Sustainable development, UNFCCC, D63, F53, N4, Q01, Q54

## Abstract

Until the late 1990s, developing countries had perceived the pursuit of development as coming into conflict with the mitigation of climate change. Research showed that mitigation and development can go hand in hand, giving rise to the co-benefits approach. In this paper, the relationship between aiming for development and aiming for climate change mitigation is analyzed from the perspective of the developing country India. While industrialized countries prefer the approach of co-benefits of mitigation, developing countries tend to follow the development-first paradigm with mitigation co-benefits, as a literature and document study show. India had a long way to come from the notion that mitigation was threatening economic growth to adopting the co-benefits approach. The paradigms of “differentiated responsibilities” and of having a right to emit as much as the industrialized countries are deeply rooted. This is also shown by India’s reaction to the current economic crisis caused by the COVID-19 pandemic.

## Introduction

Developing countries are striving for development up to the standard of living in industrialized countries. Economic growth and poverty reduction are therefore the first priorities in policy-making (Ürge-Vorsatz and Herrero 2012). Closely related to this is the increase in energy demand due to higher economic output and population growth. The International Energy Agency (IEA) estimates that if policies remain unchanged, India’s energy demand will double by 2040 (IEA 2020a). If there is no change in the development path, the increasing energy demand and population growth will lead to higher emissions of greenhouse gases (GHG). The GHG emissions of a country are positively dependent on carbon intensity, energy intensity of the gross domestic product (GDP), GDP per capita, and on population (Parikh and Parikh 2002). It is very likely that the GDP and population will continue to rise in developing countries. From 1990 to 2018, India’s population grew by 55% while the GDP per capita has increased 4.5-fold (World Bank 2020). Without an adjustment of policy objectives, emissions will inevitably rise if there is no massive technological progress (Parikh and Parikh 2002).

Climate change harms all countries. However, it is often the developing countries that are affected severely by the consequences due to their geographical conditions or dependence on climate-sensitive sectors like agriculture (Leichenko and Silva 2014). Climate change can therefore seriously constrain their development objectives in the areas of poverty alleviation, access to clean water and energy, food security, etc., and thus jeopardize the future prosperity of developing countries (Beg et al. 2002). Consequently, the mitigation of climate change is another policy objective for developing countries that has become increasingly important over the years. Such policies can have negative impacts on development targets, especially in the short term, which is why governments are often unambitious about taking mitigation measures (Pearce 2000). On the one hand, negative effects can result directly from mitigation policies; for example, the promotion of renewable energies can lead to higher electricity prices and thus come in the way of poverty alleviation. On the other hand, there are opportunity costs, since financial resources spent on mitigation cannot be invested in development measures. So, mitigation policy is closely linked to development policy, and it is necessary to find strategies that are not contrary to the objectives. An optimal policy would address and positively reinforce both. In other words, it would include measures which serve one objective and simultaneously benefit the other objective.

Industrialized countries have the incentive to emphasize the positive link between development and mitigation goals, as this could make climate protection more attractive for developing countries. However, developing countries like India are reluctant to follow the approach of co-benefits of mitigation because of fears that co-costs, which slow down development, might predominate (Jakob and Steckel 2014). In the area of climate financing,^1^ on the other hand, the link could be valuable for developing countries, as it offers the opportunity to achieve development goals through financial support of mitigation by industrialized countries.

The co-benefits approach has become increasingly relevant to international negotiations over the years and influenced decisions in climate finance. Whether mitigation of climate change or development goals are of greater importance is a recurring concern in the design of international agreements and climate financing instruments. In this paper, the relationship between aiming for development and aiming for mitigation of climate change is analyzed from the perspective of India as a developing country. For this purpose, the national climate policy is presented and placed into the context of the co-benefits approach. By means of a literature and document study, the development of the conflict of interest is analyzed from the beginning of climate policy activity. Thus, the research questions of this paper are (1) what is the relationship between aiming for development and aiming for climate change mitigation in India’s policy and (2) what influence does the conflict of interest between development and climate change mitigation have on international climate policy.

The study examines development and climate policy strategy papers and action plans of the Indian government since 2006, as well as international climate policy documents including the IPCC Assessment Reports since 2001 and the outcomes and agreements of the United Nations Framework Convention on Climate Change (UNFCCC) Parties (Conferences of the Parties) (COP) since the UNFCCC was developed in 1992. The content of the policy documents was examined for linkages between development and climate policy and for references to the term “co-benefits”. In the next section, we will look at the evolution of the co-benefits approach, demonstrating the ambivalent relationship between mitigation and development using examples from India. This is followed by a presentation of India’s political stance on mitigation and development objectives, and the meaning of the co-benefits approach in the developing country’s policy-making. The paper shows if and when India’s policies have moved more toward the mitigation objective. The last section concludes.

## Linking mitigation and development

The link between sustainable development and climate change mitigation is interpreted as co-effects that arise either from climate policy measures or from development policy measures and, simultaneously have an impact on the respective other policy area (Cohen et al. 2021).

Not least since the UN countries' agreement on the Sustainable Development Goals (SDG), the link between policies to achieve sustainable development goals and climate change policies has been intensively discussed (see, e.g., Bizikova et al. 2007; Laukkonen et al. 2009; Cohen et al. 2021).

Developed countries are trying to reinforce the positive interaction of both policies to encourage developing countries to join global climate change agreements (see, e.g., Bollen et al. 2009; Steward et al., 2013).

The most promising policy approaches are those that leverage natural synergies between climate action and development priorities to advance both goals simultaneously. Many of these synergies are e.g. in energy efficiency, renewable options and education and awareness (IPCC, 2007:702).

A link between mitigation and development has evolved gradually and was not directly implicated in the emerging climate change discourse in the late 1980s. The policy and research areas were treated rather separately due to major conceptual and methodological differences (see Cohen et al. 1998). Since the late 1990s, the interrelationship and the associated concept of co-benefits has been increasingly discussed in science and politics (Mayrhofer and Gupta 2016).

The participation of developing countries in international climate policy became important due to their rapidly growing emissions. India, for example, is one of the main emitters of CO2, accounting for about 7% of the annual global CO2 emissions (Our World in Data 2020). Among the Asian countries, only China emits more. The high relevance of developing countries in the abatement of GHG led to a strengthening of the linkage between mitigation and development to motivate them to take more action in climate policy (Najam et al. 2003).

From 2001 onwards, there was a sharp increase in publications on co-benefits, which is certainly also triggered by the study “IPCC Special Report on Emissions Scenarios (SRES)” on the relationship between mitigation and development in 2000/2001 (Bizikova et al. 2007). The Intergovernmental Panel on Climate Change (IPCC) is a directional body for international climate policy-making. At irregular intervals, it publishes its comprehensive “Assessment Reports”. They allow an insight into how dealing with climate chance has developed over the years and where the beginnings of a more integrated approach are. Sustainable development became a real issue in the Third Assessment Report, published in 2001 (Najam et al. 2003). The report demonstrates that the reduction of GHG emissions, neglecting sustainable development effects, is not the right path. Instead, mitigation is discussed from three different perspectives: cost-effectiveness, equity, and sustainable development. In contrast to the previous reports, which focused on cost-effectiveness of mitigation, the Third Assessment Report also takes into account development aspects (IPCC, 2001).

The Fourth Assessment Report, published in 2007, emphasizes “making development more sustainable” (IPCC, 2007:695). Although an ambivalent relationship between mitigation and development is reported, it clearly states the close connection between the two objectives. From then on, at the latest, the interaction of mitigation and sustainable development became central, which is also reflected in the dedication of an own chapter on this topic (Chapter 12: Sustainable Development and Mitigation). In the Fifth Assessment Report, published in 2014, the attention is put on adaptation on climate change and equity considerations (Byravan et al. 2017). The interaction between sustainable development and mitigation is granted and is declared as a basic concept for the whole report: “Sustainable development, a central framing issue in this Assessment Report, is intimately connected to climate change” (IPCC, 2014:287). With the 2030 Agenda adopted in 2015, the countries of the United Nations committed themselves to 17 Sustainable Development Goals (SDG). The guiding principle of the 2030 Agenda is to enable people around the world to live in human dignity while at the same time preserving the natural foundations of life in the long term (UN, 2015). The 2030 Agenda defines global goals taking into account social, environmental and economic aspects. Goal 13 is “Take urgent action to combat climate change and its impacts”. Thus, Mitigation of Climate Change ranks equally with the other goals.

The most recent IPCC report from 2022 also follows this direction. It not only emphasizes the inseparability of development and mitigation, but also refers to the SDGs: “There are ever closer linkages between climate change mitigation, development pathways and the pursuit of sustainable development goals. […] Climate change mitigation framed in the context of sustainable development, equity, and poverty eradication, and rooted in the development aspirations of the society within which they take place, will be more acceptable, durable and effective” (IPCC, 2022:67). In this report, at the latest, it becomes clear that mitigation of climate change can only be addressed jointly with sustainable development goals (also see Stahlke et al. 2021).

By strengthening the link between mitigation and development through the IPCC and at the insistence of developing countries (Najam et al. 2003), this could no longer be ignored in national and international policy-making. Nevertheless, it is unclear how exactly the relationship is to be structured. Due to the different interests of industrialized and developing countries, the prioritization of the global reduction of GHG emissions and of national development is varying.

### The co-benefits approach

The co-benefits approach is not a rigid policy but rather an idea that combines the pursuit of mitigation with other non-climate-specific goals (Mayrhofer and Gupta 2015). In the literature, a common definition of co-benefits does not exist (Karlsson et al. 2020). There are major differences in the interpretation and in the first and second policy objectives, that is, which of them is to be  more focused. Climate protection can be the main addressee as well as a co-benefit of other political measures especially in a development context.

Mayrhofer and Gupta (2016) identify three paradigms: development-first, climate-first, and seeking for synergies. Depending on the interests and perspectives taken (national/international, developed/developing country, etc.), policies are preferred that aim at mitigation or development with additional benefits for the other. In an initial framing paper from 2013, the Green Climate Fund (GCF) also dealt with this issue by mentioning two options: mitigation as the main focus with developmental co-benefits, or a strategy that maximizes co-benefits and only indirectly seeks mitigation (Winkler and Dubash 2016).

Some authors examine policies that address both goals equally. For example, Dubash et al. (2013) practice a holistic analysis of co-benefits, treating and assessing both sustainable development and mitigation as equivalent policy objectives. The other direction is to focus on development goals that can have subordinate mitigation effects (see, e.g., Metz et al. 2002; Michaelowa and Michaelowa 2007; Eyckmans et al. 2016).

The co-benefits approach can be used to mainstream climate protection into other policy areas (Mayrhofer and Gupta 2015). Developing countries are often hesitant to implement climate-specific measures, which is why following the path of development taking climate effects into account is a more feasible option for them (IPCC, 2007:695). If political measures become more sustainable, co-benefits for mitigation can be achieved by pursuing the development goal. However, from an international perspective, climate-specific measures are interesting. Most studies, at least those on climate change, deal with the approach of co-benefits of mitigation, that is, measures aimed at mitigation and generating co-benefits that serve the development goal (IPCC 2007:695ff) (see, e.g., Pittel and Rübbelke 2008; Nemet et al. 2010; Karlsson et al. 2020). The resulting co-benefits that help to achieve the development goal can be used to make a climate-specific measure more acceptable.

For a more detailed review of the emergence and use of the term “co-benefits” in science and politics, see, for example, Mayrhofer and Gupta (2016), Buchholz et al. (2020), and Helgenberger et al. (2019).

### Examples of conflicting interests in India’s national policies

India as a developing country, the world’s third-largest producer of GHG emissions, and as a country severely affected by climate change provides numerous examples of the complex interactions of mitigation and development. In the best case, a policy measure can address both at the same time or even strengthen each other. The following example demonstrates such a situation.

About 60% of the Indian population, especially in rural areas, used traditional cooking stoves in 2016 (IEA, 2018, 2020b). The burning of coal or biomass like wood or cow dung causes emissions that are harmful to the climate and health. The promotion of alternative cooking options could provide benefits for many different policy targets. In 2016, the Indian government launched the program “Pradhan Mantri Ujjwala Yojana” to supply 50 million households living below the poverty line with liquefied petroleum gas (LPG) through subsidies (Swain & Mishra 2020). This should reduce GHG emissions and indoor pollution caused by traditional cooking, empower women, boost the economy, and improve living conditions in rural areas (Sahoo et al. 2018). In the Indian state Odisha, for example, LPG connections increased from 12 to 33% in 2017 (Sahoo et al. 2018). A study conducted in this state, characterized by high poverty, found that the initiative actually led to women being better off socially and economically. Since they no longer have to collect firewood or make cow dung cake, they now have more time for childcare and economic activities, for example (Swain and Mishra 2020). The results of this study indicate that a political measure can both reduce GHG emissions and contribute to important development goals such as poverty reduction and economic growth. However, there are also measures to prevent GHG emissions which have negative implications for the development of a country. Such effects have been observed for some hydropower projects, for example.

The West Himalayan region has great potential for hydropower projects due to the abundance of water and the hilly landscape. As the demand for electricity is strongly on the rise, hydropower projects can contribute to a climate-friendly growth of the region (Sharma and Thakur 2017). A study in the state of Jammu and Kashmir by Sharma and Thakur (2017) on a project that also generates carbon credits under the Kyoto Protocol found positive development effects, such as the improvement of transportation and communication facilities. However, the case study also identified considerable negative effects that increase poverty for some population groups. The examined project caused displacing of people, danger of waterlogging, salinity, and acquisition of agricultural land. Another study, also conducted in this region, found increasing deforestation, loss of flora and fauna, soil erosion, and the drying up of natural water as a result of the tunneling activity for hydro projects (Sharma and Rana 2014). In addition, construction and operation can pose a threat to the people living in this region, resulting in a high number of deaths caused by landslides, water inrushes in tunnels, and blasting (Kumar and Katoch 2017). Hydropower projects can thus prevent GHG emissions, but might harm sustainable development more than possibly other energy sources would. So, climate protection can have co-benefits for a certain development goal, such as economic growth, but also work against another, such as poverty alleviation. These conflicting goals often relate to distributive justice. In many cases, only certain population groups benefit, while others bear the costs. Many growth-stimulating policies benefit only the already propertied population, while the lower segments of the population do not participate or even suffer, which can increase poverty (see, e.g., Büchs et al. 2011; Yenneti and Day 2016). The fact that the positive effects often do not outweigh the negative ones in large hydropower projects can be seen in the Narmada Bachao Andolan movement. This is a social movement initiated by indigenous people, farmers, and environmentalists (Mallick 2021). It started in 1985 and has its origins in the protest against large dam projects across the Narmada River (Rekha et al. 2022). It quickly grew into an international protest against the destruction of land, biodiversity, and resettlement (Mallick 2021). Thus, the adverse effects of types of mitigation projects can be considerable and even rather detrimental to the local people and the development of the country.

However, it is not only climate protection measures that face this problem of addressing different development goals in a contradictory way. In addition to the numerous measures that promote development that are potentially harmful to the climate, there are also those that simultaneously inhibit other development goals. To stimulate growth in India, many state-owned coal mines were awarded to private companies during the liberalization of the mining industry in 1991 (Singh and Kalirajan 2003). The states Jharkhand and Odisha in particular have large coal reserves. The privatization not only brought income for the mining industry, but also created jobs and infrastructure development (Mishra 2009). Besides the negative consequences of coal for climate change, this measure also had other developmentally restrictive effects. The sale dispossessed tribal people and other landowners without adequate compensation, which is contrary to the development goal of poverty reduction and distributive justice (see, e.g., Lahiri-Dutt et al. 2012; Bedi 2013). This also caused serious water, air, and noise pollution, which in turn impaired the quality of life of the local population (Mishra 2009).

The interaction between mitigation and development is therefore complex, since not only can the two hinder or encourage each other, but they can also lead to conflicting different development goals. The co-benefits approach could take this into account by also considering possible co-costs in an evaluation of policy measures (see, e.g., Ruth 2011; Ürge-Vorsatz et al. 2014).

## India’s balancing act

This section will look at the rise of India’s climate policy. It shows how India has adopted the co-benefits approach, starting from an attitude that just the main causers of climate change, the industrialized countries, have to reduce GHG emissions. It is a common dilemma that climate protection measures, which are desirable from a global point of view, are considered to be of little use at the local level. There is also an incentive for measures that are conducive to development in the country to be communicated internationally as climate protection measures (see, e.g., Mayrhofer and Gupta 2015).

The focus in this section is on the relationship between the mitigation of climate change and development objectives, and on the extent to which these objectives are seen conditional on or contrary to each other. Hence, both international and national objectives will be identified.

### Early climate policy

India’s climate policy has gone through various phases, with the period around 2007 and then again in 2015 probably marking the high points in its climate activities. Article 3, Paragraph 1 of the UNFCCC, which was adopted in 1992, states: “The Parties should protect the climate system […] on the basis of equity and in accordance with their common but differentiated responsibilities …”. This principle of “differentiated responsibilities” reveals a disparity between developing and industrialized countries in carrying the burden of mitigation of climate change. Based on this principle, India expected to receive financial support for their climate activities from the global North, since climate change is mainly due to past emissions from the latter (Atteridge et al. 2012). This manifested paradigm determined India’s climate policy. Another principle that India has adhered to is that of equity, in the sense of having a right to emit as much as the industrialized countries (Bidwai 2012). Equality, however, should not be measured in terms of the absolute emissions of countries, but rather in terms of per-capita emissions (Shukla and Dhar 2011). In India, the per-capita CO2 emissions have increased 1.6-fold between 1990 and 2017 (Our World in Data 2020). Nevertheless, in 2017 it only had per-capita emissions of 1.8 tons, while, for example, Germany emitted 9.7 tons CO2 per capita (Our World in Data 2020). Since India’s per-capita emissions would be far lower than those of industrialized countries and many developing countries, India did not see itself as responsible for entering into emission reduction commitments (Jayaram 2018). Instead, it insisted on the right on equal per-capita emissions.

These two principles are also reflected in the National Environment Policy (NEP). In 2006, the NEP was introduced to set the framework for India’s environmental policy. It concentrates on local environmental degradation, and climate change is only a small part of it. While India recognizes that climate change is a global problem that poses a threat to India and other developing countries, it sees the responsibility as lying with the industrialized countries due to its low per-capita emissions (only 4% of the USA’s and 8% of Germany’s levels in the year 1994, as the NEP states) (Government of India 2006:42). India’s understanding of equity is expressed by: “Equal per-capita entitlements of global environmental resources to all countries” (Government of India 2006:43). The NEP demonstrates the subordinate position of mitigation by emphasizing India’s “[o]ver-riding priority of the right to development” (Government of India 2006:43).

India’s early climate policy was also determined by the assumption that mitigation slows down economic growth and must therefore be weighed against national goals such as poverty reduction (Atteridge et al. 2012). It was feared that India’s development would be harmed, especially by energy-saving measures, as this could slow down rural electrification (Parikh and Parikh 2002). As long as India saw its development of economic prosperity threatened by mitigation actions, voluntary measures were only rarely taken (Sathaye et al. 2006). The NEP explicitly mentions the economic costs that would arise from taking mitigation measures: “… abatement of GHGs, would involve significant economic costs” (Government of India 2006:41). Therefore, in the first phase of climate policy, mitigation was seen more as a threat to economic growth (Isaksen and Stokke 2014), and possible development co-benefits were not considered.

### The national action plan on climate change (NAPCC)

Starting in 2007, India changed its climate policy, which eventually led to the adoption of the National Action Plan on Climate Change (NAPCC) in 2008. The climate debate was being increasingly taken up nationally (Dubash 2013). The equity principle became gradually less relevant due to three other aspects that influenced or fueled climate policy: The “hiding behind the poor” debate,^2^ the growing need for climate adaptation measures, and the existing energy insecurity caused by economic growth combined with a lack of supply (Dubash 2013). So, on the one hand, India became increasingly aware of its vulnerability and the danger this posed to its economic growth. On the other hand, it began embracing mitigation as an opportunity to achieve its development goals. For example, it realized that creating a solar manufacturing hub could boost its economy (Mayrhofer and Gupta 2015). India also faced increasing international pressure to take climate action in order not to endanger other foreign co-operations, such as the Indo–US nuclear deal or relations with China.

The NAPCC sets the framework for India’s actions against climate change. It outlines “eight missions” that include both mitigation and adaptation, with an enhanced focus on solar power and energy efficiency. Probably the two most important missions are the National Solar Mission, with the goal of deploying 20 GW of grid-connected solar power by 2022 (later extended to 100 GW [Government of India 2020a]) and the National Mission of Enhanced Energy Efficiency, which, among other things, resulted in the introduction of the Perform Achieve and Trade (PAT) system. This is a market-based instrument that encourages large energy consumers in the industry to trade energy-saving certificates and is the first energy efficiency trading scheme adopted by developing countries (Virmani and Rao 2015).

Even if the NAPCC demonstrates India’s steps toward greater climate action, its development goals determine the agenda. Almost all of the stated principles are related to development objectives, such as poverty alleviation, economic growth, energy security, and technological progress, and are closely linked to its need to adapt to climate change. Nevertheless, the NAPCC is an important step, indicating that India has changed its attitude that mitigation and development are contradictory. Instead, it recognizes that pursuing development could have co-benefits for the climate. This was a turning point in India’s climate policy. Especially the acknowledgement of the close link between mitigation and energy security concerns has paved the way for more climate action (Dubash 2013). Co-benefits were evident in areas such as energy efficiency, urban development, and water supply (Dubash et al. 2018). There was a shift from considering mitigation as a threat to the acceptance of the co-benefits approach. However, the country’s interpretation is characterized by the development-first attitude. The NAPCC indicates that effects on sustainable development are not co-benefits from many of the introduced measures, but the positive effects on the climate result as co-benefits of measures that actually promote development. The statement that “The National Action Plan on Climate Change identifies measures that promote our development objectives while also yielding co-benefits for addressing climate change effectively” (Government of India, 2008:2) at the beginning of the NAPCC demonstrates India’s priority.

At the state level, this is even more evident. The NAPCC requests all Indian states to submit “State Action Plans on Climate Change” (SAPCC), which should present state-level climate activities (Chandel, 2016). However, these documents are often agendas for social and economic development with possible co-benefits for the climate (Atteridge et al. 2012). In addition, the implementation of measures outlined in the SAPCCs often fails due to a lack of co-ordination between central and state governments with respect to responsibility and funding (Pahuja et al. 2014). India balances between addressing the development and adaptation needs of the states, and the increasing international motivations to mitigate climate change (Atteridge et al. 2012).

In addition, the NAPCC is still based on India’s principles of “differentiated responsibilities” (Dubash and Ghosh 2019) and equity: “We are convinced that the principle of equity that must underlie the global approach must allow each inhabitant of the earth an equal entitlement to the global atmospheric resource” (Government of India 2008:2). Insisting on the equity principle, India also appeases industrialized countries by claiming: “… India is determined that its per capita greenhouse gas emissions will at no point exceed that of developed countries even as we pursue our development objectives” (Government of India 2008:2). Conflicts of interest are evident both at the state level and at the international level. Despite holding on to these notions, the NAPCC is an important step in India’s climate policy, as it acknowledges that development and mitigation are closely linked and co-benefits can be realized.

### Preparatory actions in the run-up to the COP 15 in Copenhagen

India took the next important step in its climate policy in 2009. India teamed up with three emerging economies—Brazil, China, and South Africa—to jointly represent their interests at the Conference of the Parties (COP) 15 in Copenhagen. Joining this alliance initiated by China had also strategic reasons, as China is an important trading partner and as a neighbor a relevant factor for India’s national security (Atteridge et al. 2012). This alliance of the four so-called “BASIC countries” gave India more power in the negotiations with the industrialized countries, as the emerging economies together are responsible for about one-third of global emissions from energy use and are therefore a significant player in combating climate change (Shukla and Dhar 2011). In negotiations that preceded the COP 15, India voluntarily committed to reduce its emissions intensity of GDP by 20–25% compared to 2005 by 2020 (Jayaram 2018).

Nevertheless, the resulting Copenhagen Accord was not very successful in resolving climate action. Instead of binding commitments or more far-reaching international co-operation, countries only made general statements regarding their climate targets (Hunter 2010). This and the emphasis on sustainable development in the Copenhagen Accord indicates the strong negotiating power of the BASIC alliance. Again, the principle of “differentiated responsibilities” (Article 1, Copenhagen Accord) and equity considerations (Article 2, Copenhagen Accord) were laid down. In addition, it was also recognized that climate protection taken by developing countries is subordinate to their development goals: “… social and economic development and poverty eradication are the first and overriding priorities of developing countries …” (UNFCCC, 2009). This virtually gave developing countries permission for their development-first attitude. Therefore, it can be argued that the Copenhagen Accord was disappointing for the global goal of mitigation, but it was satisfying for India, the other BASIC countries, and the rest of the developing world.

### The internationally committed plan against climate change

The COP 21 in Paris in 2015 marks a watershed in international climate policy because India and all parties of the UNFCCC agreed to limit the man-made global temperature increase caused by the greenhouse effect to 1.5 °C compared to pre-industrial levels. Around this time, India took an active part in international climate policy (Isaksen and Stokke 2014). India founded the International Solar Alliance (ISA), which has 122 member countries to date and aims to promote the use and quality improvements of solar energy (IEA 2018). Moreover, the government also set the target of installing 175 GW of renewable energy by 2022 (Mohan 2017). This is also due to its recognition that mitigation has valuable co-benefits for its energy goals.

As India is heavily dependent on crude oil imports, a shift toward renewable energies also helps to enhance energy security. India imports 80% of its crude oil needs (Dalei and Gupta 2020). Crude oil covers 20% of the total energy supply (ISA, 2020a). This dependence, mainly on countries in the Middle East and Africa (Tiewsoh et al. 2017), could be reduced by supporting other energies, such as renewables. The pursuit of energy security is likely to have contributed to India’s commitment to expand climate-friendly energies. The adoption of the co-benefits approach, and the growing understanding and media coverage of the causes and effects of climate change have influenced India’s climate activities (Jayaram 2018). In 2014, this even resulted in the renaming of India’s “Ministry of Environment and Forests” as “Ministry of Environment, Forest and Climate Change.”

With the signing of the Paris Agreement and the associated commitment to pursue its Nationally Determined Contribution (NDC) submitted in the run-up to COP 21, India agreed to contribute jointly to the global goal of climate change mitigation. For the first time, India and other developing countries made an international binding commitment to fulfill mitigation targets. The NDC, which describes the climate targets and action plans for 2021–2030, is the second key document for India’s climate policy alongside the NAPCC. The NDC proposes three quantitative pledges: emission intensity is to be reduced 33–35% by 2030 from 2005 levels (Pledge 3), the share of non-fossil fuel-based energy is to be increased to 40% (Pledge 4), and a carbon sink of 2.5–3 billion tons of CO2eq is to be created through afforestation (Pledge 5) (Government of India 2015:29).

Despite these commitments, India has not fully renounced its principle of equity: “Our objective is to establish an effective, cooperative and equitable global architecture based on climate justice and the principles of Equity and Common But Differentiated Responsibilities and Respective Capabilities, under the UNFCCC” (Government of India 2015:3). So, the paradigm of “differentiated responsibilities” is also valid. This is reflected in India’s making its climate targets dependent on financial and technological support from other countries: “… and being sanguine about the unencumbered availability of clean technologies and financial resource from around the world, …” (Government of India 2015:29).

India clarifies which goal it pursues primarily by stating: “… where eradication of poverty is one of the foremost priorities” (Government of India 2015:4f). However, the NDC also indicates a small shift toward a greater emphasis on mitigation: “… to exploit the co-benefits of addressing climate change along with promoting economic growth” (Government of India 2015:7). Although this statement suggests that a climate-first strategy is also possible, the NDC in general and India’s policy actions continue to be focused on the pursuit of development. In the literature, India’s NDC is assessed as disappointing. The commitments are seen as too unambitious and inconsistent with domestic policy (Mohan and Wehnert 2018). It refers to the NAPCC, lists a large number of measures and policies that are already in place or planned, and shows little progress (Dubash and Ghosh 2019). Even though efficiency improvements are planned, India intends to stick to its coal-based energy generation: “… coal will continue to dominate power generation in future” (Government of India 2015:10). Most of the measures are described as climate protection, but with the condition that development is guaranteed. Even if measures serve mainly the purpose of mitigation, these are always strongly linked to development goals (Dubash and Ghosh 2019).

As in the NAPCC, the co-benefits approach is also adopted in the NDC. While the NAPCC tends to be more a national development agenda with co-benefits for the climate, the NDC is designed for international communication and is thus actually primarily intended to pledge climate targets. India’s dichotomy between national and international interests is apparent here. The co-benefits approach is used to present a good image abroad and promote international relations, while at the same time communicating domestically that development goals are the first to be achieved (Mayrhofer and Gupta 2015).

So, the measures to mitigate climate change described in the NDC do not greatly exceed those in the NAPCC, are dependent on financial support from developed countries, and the paradigm of equity still applies. These factors indicate that India maintains its development-first attitude. However, a small progress in shifting the focus more to mitigation with development co-benefits is apparent in the NDC. How India’s stance on the relationship between climate and development will develop further will possibly be reflected in an updated and more ambitious NDC as the first five-year cycle of the NDC ends.

### Policy goals during a crisis

The difficulty of reconciling national development goals with the international fight against climate change became obvious in 2020. The COVID-19 pandemic has severe economic consequences worldwide. In India, repeated lockdowns have massively reduced economic activity, which also has consequences for the energy sector (TERI, 2020). It is estimated that the GDP fell by 9.3% between April and June 2020 (Statista 2020a). In reaction to this situation, the Indian government launched an action plan in mid-May. Under the program “Atmanirbhar Bharat Abhiyan”, meaning “Self-reliant India Mission”, wide-ranging economic assistance is promised. The national plan covers five areas—economy, infrastructure, system, vibrant demography, and demand (Government of India 2020b). Under the headline “New Horizons of Growth”, policy reforms are presented, especially in the coal sector.

In 2017, coal accounted for 44% of total energy supply, of which about 13% was imported (IEA, 2018). India thus imports a share of its main energy sources oil (80%) and coal (13%). The Atmanirbhar Bharat Abhiyan aims to reduce dependence on other countries and strengthen the economy. Given that India has one of the largest coal reserves in the world with 100 billion metric tons (Statista 2020b), the Indian government has decided to support the national coal industry. To this end, it will auction around 50 coal mines to the private sector and commercialize the mining industry. The goal is to produce 1 billion tons more coal by 2023/2024. In addition to this measure, more than 6 billion dollars will be invested in the expansion of coal infrastructure (Government of India 2020b). This means that not only will climate-damaging emissions be released, but also important carbon sinks will be destroyed by deforestation (see, e.g., Ranjan et al. 2020). In addition, biodiversity will be threatened and indigenous population groups displaced (Energiezukunft 2020). The plan indicates that India expects more and faster benefits from coal production, such as economic growth and independence, than from renewable energies. It is not only climate commitments that are sacrificed, but also other development goals such as poverty reduction or the health of the population. Making things even more difficult is the fact that the coal lobby still holds great sway. This also shows how India is trying to position itself between regional, national, and international interests. A crisis can quickly lead to shifts in policy goals and a return to protecting the country's own economic stability.

### Updates from the latest COPs

The perceived equity of climate finance instruments influences the willingness of states to participate in international agreements. The design or institutional arrangement of climate finance mechanisms is therefore a key factor. It must be perceived as equitable by all countries. That this is a formidable challenge is demonstrated in the recent international climate policy. The Climate Conference in Glasgow COP 26 in November 2021 resulted in the “Glasgow Climate Pact”. With this, the Parties referred for the first time to the phase-out of coal-fired power generation and the abolition of inefficient subsidies for fossil fuels, and again emphasized the 1.5 °C target (United Nations and UK Government, 2021). However, the result was diluted at the last minute, as China, India, and Iran vetoed it. Instead of “phase-out” for coal and subsidies, the Glasgow Climate Pact now only says “phase down”. India illustrated once again that climate justice is a top priority. However, in a speech by the Prime Minister of India, the goal of net-zero emissions by 2070 was stated. This was the first time the country announced its intention to become climate-neutral, even though 2070 has been criticized by other countries as being far too late (Hasan 2022).

At the recent COP 27 conference in Sharm el-Sheikh, China and India, which together with the USA are among the largest CO2 emitters, did not even attend. The conference was hardly successful in the area of mitigation of climate change. However, a new fund for compensation of loss and damage was agreed (see United Nations 2022). Even though it is still unclear for which countries and by whom the funds will be made available, after decades of demands from vulnerable states, the creation of the fund is an important signal of solidarity.

It can be expected that the vulnerability of a state has an increasing impact on international negotiations. The allocation of financial resources can be an effective strategy to affect mitigation actions of developing countries.^3^ The future will show how the countries’ effort to implement climate protection measures can be influenced by the allocation of aid.

## Conclusion

While the Kyoto Protocol, adopted in 1997, focused on rapid emission reductions, the Paris Agreement, sealed in 2015, shows a stronger focus on the interaction between mitigation and sustainable development. This link between the two policy objectives did not emerge abruptly but is the result of years of development and international climate negotiations.

In India, too, the move from the conviction that the two goals are incompatible to the understanding that they can mutually benefit each other only became apparent around 2008 in its NAPCC. But even though the co-benefits approach has seen large adoption, India still adheres to its principles of equity and “differentiated responsibilities”, as the NDC demonstrates.

As the literature and document study indicates, industrialized countries seek to emphasize the link to development in order to motivate developing countries to participate in international climate change mitigation agreements and reduce their GHG emissions. Developing countries, however, seem convinced that industrialized countries, being the principal agents of climate change, are under obligation to mitigate, and they are therefore striving to achieve as few restrictions on their emissions as possible with the maximum link to their development goals (Najam et al. 2003). Negotiating the balance between the two policy objectives is always present at international climate conferences. In this respect, the Paris Agreement can be seen as a successful outcome of negotiations between industrialized and developing countries. On the one hand, for the first time, developing countries have made internationally binding commitments to reduce their GHG emissions; on the other hand, industrialized countries have stated that the consideration of development goals is indispensable. Article 2 of the agreement stipulates that the common goal of keeping global warming below 2 degrees be addressed “in the context of sustainable development” (UNFCCC 2015).

Despite the strong presence of the development target in the Paris Agreement, it is likely that, given their reduction commitments, the attitude of developing countries will shift further from development-first to climate-first. However, the current crises can halt this process, revealing just how fragile the balance between mitigation and development is, as the last COPs also showed.

## Data Availability

No.

## Footnotes

1 It should be noted that climate finance, i.e., transfers from industrialized countries to developing countries, can be problematic, e.g., due to debt traps, or the donorship-syndrome (see, e.g., Hourcade et al. 2021; Browne 2022).

2 The “hiding behind the poor” debate was triggered by a Greenpeace report on the positive link between emissions and wealth in India (see, e.g., Chakravarty and Ramana, 2011).

3 Adaptation aid can depend on how the mitigation behavior of recipient countries changes as a consequence (Mukherjee et al. 2022).

## Code availability

No.

## References

[CR1] Atteridge A, Shrivastava KM, Pahuja N, Upadhyay H (2012). Climate policy in India: what shapes international, national and state policy?. Ambio.

[CR2] Bedi HP (2013). Environmental Mis-assessment, development and mining in Orissa India. Dev Change.

[CR3] Beg N, Morlot JC, Davidson O, Afrane-Okesse Y, Tyani L, Denton F, Sokona Y, Thomasc JP, La Rovere EL, Parikh JK, Parikh K, Rahman AA (2002). Linkages between climate change and sustainable development. Climate Policy.

[CR4] Bidwai P, Hällström N (2012). Climate change, equity and development—India’s dilemmas. What next.

[CR5] Bizikova L, Robinson J, Cohen S (2007). Linking climate change and sustainable development at the local level. Climate Policy.

[CR6] Bollen J, Guay B, Jamet S & Corfee-Morlot J (2009). Co-benefits of climate change mitigation policies: literature review and new results. *Economics Department Working Papers No. 693*. OECD, ECO/WKP(2009)34.

[CR7] Browne K (2022). Rethinking governance in international climate finance: structural change and alternative approaches. Wires Clim Change.

[CR8] Buchholz W, Markandya A, Rübbelke D, Vögele S, Buchholz W, Markandya A, Rübbelke D, Vögele S (2020). Analysis of Ancillary Benefits of Climate Policy. Ancillary benefits of climate policy: new theoretical developments and empirical findings.

[CR9] Büchs M, Bardsley N, Duwe S (2011). Who bears the brunt? Distributional effects of climate change mitigation policies. Crit Soc Policy.

[CR10] Byravan S, Ali MS, Ananthakumar MR, Goyal N, Kanudia A, Ramamurthi PV, Srinivasan S, Paladugula AL (2017). Quality of life for all: a sustainable development framework for India's climate policy reduces greenhouse gas emissions. Energy Sustain Dev.

[CR11] Chakravarty S, Ramana MV, Dubash NK (2011). Hiding behind the poor debate: a synthetic overview. Handbook of climate change and India: development, politics and governance.

[CR12] Chandel SS, Shrivastva R, Sharma V, Ramasamy P (2016). Overview of the initiatives in renewable energy sector under the national action plan on climate change in India. Renew Sustain Energy Rev.

[CR13] Cohen S, Demeritt D, Robinson J, Rothman D (1998). Climate change and sustainable development: towards dialogue. Glob Environ Chang.

[CR14] Cohen B, Cowie A, Babiker M, Leip A, Smith P (2021). Co-benefits and trade-offs of climate change mitigation actions and the sustainable development goals sustain. Prod Consum.

[CR15] Dalei NN, Gupta A, Gupta A, Dalei N (2020). India’s crude oil consumption: empirical estimations and future projections. Energy, environment and globalization.

[CR16] Dubash NK (2013). The politics of climate change in India: narratives of equity and cobenefits. Wires Clim Change.

[CR17] Dubash NK, Ghosh S, Dubash NK (2019). National climate policies and institutions. India in a warming world.

[CR18] Dubash NK, Raghunandan D, Sant G, Sreenivas A (2013). Indian climate change policy: exploring a co-benefits based approach. Econ Pol Wkly.

[CR19] Dubash NK, Khosla R, Kelkar U, Lele S (2018). India and climate change: evolving ideas and increasing policy engagement. Annu Rev Environ Resour.

[CR20] Energiezukunft (2020). Indien setzt auch zukünftig auf Kohlekraft. 19.08.2020. https://www.energiezukunft.eu/wirtschaft/indien-setzt-auch-zukuenftig-auf-kohlekraft/. Accessed 25 Aug 2020

[CR21] Eyckmans J, Fankhauser S, Kverndokk S (2016). Development aid and climate finance. Environ Resource Econ.

[CR22] Government of India (2006). National environment policy 2006. https://ibkp.dbtindia.gov.in/DBT_Content_Test/CMS/Guidelines/20190411103521431_National%20Environment%20Policy,%202006.pdf. Accessed 28 July 2020

[CR23] Government of India (2008). National action plan on climate change. https://archivepmo.nic.in/drmanmohansingh/climate_change_english.pdf. Accessed 28 July 2020

[CR24] Government of India (2015). India’s intended nationally determined contribution: working towards climate justice. https://www4.unfccc.int/sites/NDCStaging/Pages/All.aspx. Accessed 30 July 2020

[CR25] Government of India (2020a). National portal for renewable purchase obligation (RPO). https://rpo.gov.in/Home/Objective. Accessed 28 July 2020a

[CR26] Government of India (2020b). Aatmanirbhar Bharat Abhiyaan Phase-IV to support Indian economy in fight against COVID-19. 16.05.2020b. https://covid19.india.gov.in/document-category/ministry-of-finance/. Accessed 21 Aug 2020b

[CR27] Hasan M (2022). Climate justice and COP26: a new perspective on the climate crisis. Med Confl Surviv.

[CR28] Helgenberger S, Jänicke M, Gürtler K, Leal Filho W, Azul A, Brandli L, Özuyar P, Wall T (2019). Co-benefits of Climate Change Mitigation. Climate action: Encyclopedia of the UN sustainable development goals.

[CR29] Hourcade J-C, Dasgupta D, Ghersi F (2021). Accelerating the speed and scale of climate finance in the post-pandemic context. Climate Policy.

[CR30] Hunter D (2010). Implications of the copenhagen accord for global climate governance. Sustain Dev Law Policy.

[CR31] IEA [International Energy Agency] (2018). India: key energy statistics, 2018. https://www.iea.org/countries/india. Accessed 21 Aug 2020.

[CR32] IEA [International Energy Agency] (2020a). India 2020a: energy policy review. https://www.iea.org/reports/india-2020a. Accessed 28 July 2020.

[CR33] IEA [International Energy Agency] (2020b). Share of population with clean cooking access in the new policies scenario, 2000–2030. https://www.iea.org/data-and-statistics/charts/share-of-population-with-clean-cooking-access-in-the-new-policies-scenario-2000-2030. Accessed 21 Aug 2020

[CR34] IPCC [Intergovernmental Panel on Climate Change] (2001). Climate change 2001: mitigation. Contribution of working group III to the third assessment report of the intergovernmental panel on climate change. Cambridge University Press

[CR35] IPCC [Intergovernmental panel on climate change] (2007). Climate change 2007: mitigation of climate change. Contribution of working Group III to the fourth assessment report of the IPCC. Cambridge University Press

[CR36] IPCC [Intergovernmental Panel on Climate Change] (2014). Climate change 2014: mitigation of climate change. Contribution of working group III to the fifth assessment report of the IPCC. Cambridge University Press

[CR37] IPCC [Intergovernmental Panel on Climate Change] (2022). Climate change 2022: mitigation of climate change. Contribution of working group III to the sixth assessment report of the IPCC. Cambridge University Press

[CR38] ISA [International Solar Alliance] (2020). Background. https://isolaralliance.org/about_us_history.php. Accessed 28 July 2020

[CR39] Isaksen K, Stokke K (2014). Changing climate discourse and politics in India. Climate change as challenge and opportunity for diplomacy and development. Geoforum.

[CR40] Jakob M, Steckel JC (2014). How climate change mitigation could harm development in poor countries. Wires Clim Change.

[CR41] Jayaram D (2018). From “spoiler” to “bridging nation”: the reshaping of India’s climate diplomacy. Revue Int Et Stratégique.

[CR42] Karlsson M, Alfredsson E, Westling N (2020). Climate policy co-benefits: a review. Climate Policy.

[CR43] Kumar D, Katoch SS (2017). Dams turning devils: an insight into the public safety aspects in operational run of the river hydropower projects in western Himalayas. Renew Sustain Energy Rev.

[CR44] Lahiri-Dutt K, Krishnan R, Ahmad N (2012). Land acquisition and dispossession: private coal companies in Jharkhand. Econ Pol Wkly.

[CR45] Laukkonen J, Blanco P, Lenhart J, Keiner M, Cavric B, Kinuthia-Njenga C (2009). Combining climate change adaptation and mitigation measures at the local level. Habitat Int.

[CR46] Leichenko R, Silva JA (2014). Climate change and poverty: vulnerability, impacts, and alleviation strategies. Wires Clim Change.

[CR47] Mallick K, Mallick K (2021). Narmada Bachao Andolan (NBA): save the Narmada. Environmental movements of India: Chipko, Narmada Bachao Andolan, Navdanya.

[CR48] Mayrhofer JP, Gupta J (2015). The politics of co-benefits in India’s energy sector. Gov Policy.

[CR49] Mayrhofer JP, Gupta J (2016). The science and politics of co-benefits in climate policy. Environ Sci Policy.

[CR50] Metz B, Berk M, den Elzen M, de Vries B, van Vuuren D (2002). Towards an equitable global climate change regime: compatibility with article 2 of the climate change convention and the link with sustainable development. Climate Policy.

[CR51] Michaelowa A, Michaelowa K (2007). Climate or development: is ODA diverted from its original purpose?. Clim Change.

[CR52] Mishra PP (2009). Coal mining and rural livelihoods: case of the Ib valley coalfield Orissa. Econ Polit Wkly.

[CR53] Mohan A (2017). From Rio to Paris: India in global climate politics. Rising Powers q..

[CR54] Mohan A, Wehnert T (2018). Is India pulling its weight? India’s nationally determined contribution and future energy plans in global climate policy. Climate Policy.

[CR55] Mukherjee V, Rübbelke D, Stahlke T, Brumme A (2022). Allocation of adaptation aid: a normative theory. J Econ Stat.

[CR56] Najam A, Huq S, Sokona Y (2003). Climate negotiations beyond Kyoto: developing countries concerns and interests. Climate Policy.

[CR57] Nemet GF, Holloway T, Meier P (2010). Implications of incorporating air quality co-benefits into climate change policymaking. Environ Res Lett.

[CR58] Our World in Data (2020). CO_2_ and greenhouse gas emissions. https://ourworldindata.org/co2-and-other-greenhouse-gas-emissions. Accessed 28 July 2020

[CR59] Pahuja, N., Pandey, N., Mandal, K. & Bandyopadhyay, C. (2014). GHG Mitigation in India: an Overview of the Current Policy Landscape. Working Paper. Washington DC: World Resources Institute.

[CR60] Parikh JK & Parikh K (2002) Climate change: India’s perceptions, positions, policies and possibilities. Working Paper. OECD, Paris

[CR61] Pearce D (2000). Policy frameworks for the ancillary benefits of climate change policies. Workshop on assessing the ancillary benefits and costs of greenhouse gas mitigation strategies.

[CR62] Pittel K, Rübbelke D (2008). Climate policy and ancillary benefits: a survey and integration into the modelling of international negotiations on climate change. Ecol Econ.

[CR63] Ranjan AK, Sahoo D, Gorai AK (2020). Quantitative assessment of landscape transformation due to coal mining activity using earth observation satellite data in Jharsuguda coal mining region, Odisha, India. Environ Dev Sustain.

[CR64] Rekha, R., Advaita, R. & Anas, N. (2022). Understanding the existential crisis of environmental movements in neoliberal India. *J Polit Soc*, 14(1). https://journalspoliticalscience.com/index.php/i/article/view/13.

[CR65] Ruth, M. (2011). Managing Regional Climate Mitigation and Adaptation Co-benefits and Co-costs. In: Otto-Zimmermann K (Ed.), *Resilient Cities. Local Sustainability* (pp. 205–212). Vol 1. Dordrecht: Springer, London

[CR66] Sahoo MC, Patel P, Patel R (2018). Grassroots energy security for India’s poor and women empowerment: an assessment of Pradhan Mantri Ujjwala Yojana. J Governance Public Policy.

[CR67] Sathaye J, Shukla PR, Ravindranath NH (2006). Climate change, sustainable development and India: global and national concerns. Curr Sci.

[CR68] Sharma HK, Rana PK (2014). Assessing the impact of hydroelectric project construction on the rivers of District Chamba of Himachal Pradesh in the Northwest Himalaya, India. Int Res J Soc Sci.

[CR69] Sharma AK, Thakur NS (2017). Energy situation, current status and resource potential of run of the river (RoR) large hydro power projects in Jammu and Kashmir: India. Renew Sustain Energy Rev.

[CR70] Shukla PR, Dhar S (2011). Climate agreements and India: aligning options and opportunities on a new track. Int Environ Agreements.

[CR71] Singh K, Kalirajan K (2003). A decade of economic reforms in India: the mining sector. Resour Policy.

[CR72] Stahlke T, Some S, Mukherjee V, Rübbelke D, Leal Filho W, Luetz JM, Ayal D (2021). Co-Benefits of CDM’s Renewable energy projects in india and their contribution to SDGs. Handbook of climate change management.

[CR73] Statista (2020a). Estimated quarterly impact from the coronavirus (COVID-19) on India's GDP growth in 2020a. https://www.statista.com/statistics/1103120/india-estimated-impact-on-gdp-growth-by-coronavirus-epidemic/. Accessed 23 Aug 2020a.

[CR74] Statista (2020b). Proved coal reserves in India in 2011 to 2019. https://www.statista.com/statistics/265442/proved-coal-reserves-in-india/. Accessed 23 Aug 2020b.

[CR75] Stewart R, Oppenheimer M, Rudyk B (2013). A new strategy for global climate protection. Clim Change.

[CR76] Swain SS, Mishra P (2020). Determinants of adoption of cleaner cooking energy: experience of the Pradhan Mantri Ujjwala Yojana in rural Odisha India. J Clean Prod.

[CR77] TERI [The Energy & Resource Institute] (2020). Impact of Coronavirus on the Indian Energy Sector. https://www.teriin.org/article/impact-coronavirus-indian-energy-sector. Accessed 23 Aug 2020

[CR78] Tiewsoh LS, Sivek M, Jirásek J (2017). Traditional energy resources in India (coal, crude oil, natural gas): a review. Energy Sources Part B.

[CR79] UNFCCC [United Nations Framework Convention on Climate Change] (2009). Report of the Conference of the Parties on its fifteenth session, held in Copenhagen from 7 to 19 December 2009. Addendum. Part Two: action taken by the Conference of the Parties at its fifteenth session. FCCC/CP/2009/11/Add.1, Decision 2/CP.15. Copenhagen Accord.

[CR80] UNFCCC [United Nations Framework Convention on Climate Change] (2015). Report of the Conference of the Parties on its twenty-first session, held in Paris from 30 November to 13 December 2015. Addendum. Part Two: action taken by the Conference of the Parties at its twenty-first session. FCCC/CP/2015/10/Add.1, Decision 1/CP.21. Paris Agreement

[CR81] United Nations and UK Government (2021). COP26: the Glasgow Climate Pact. UN, Glasgow. https://ukcop26.org/wp-content/uploads/2021/11/COP26-Presidency-Outcomes-The-Climate-Pact.pdf.

[CR82] United Nations (2015). Transforming Our World, the 2030 Agenda for Sustainable Development. General Assembly Resolution A/RES/70/1. UN, New York.

[CR83] United Nations (2022). Decisions taken at the Sharm El-Sheikh Climate Change Conference. https://unfccc.int/cop27/auv. Accessed 25 Jan 2023.

[CR84] Ürge-Vorsatz D, Herrero ST (2012). Building synergies between climate change mitigation and energy poverty alleviation. Energy Policy.

[CR85] Ürge-Vorsatz D, Herrero ST, Dubash NK, Lecocq F (2014). Measuring the co-benefits of climate change mitigation. Annu Rev Environ Resour.

[CR86] Virmani A, Rao DN (2015). A study of climate change control schemes: India's pat scheme with other international climate change control schemes. Available at SSRN 2547367.

[CR87] Winkler H, Dubash NK (2016). Who determines transformational change in development and climate finance?. Climate Policy.

[CR88] World Bank (2020). Data—indicators. https://data.worldbank.org/indicator. Accessed 28 July 2020

[CR89] Yenneti K, Day R (2016). Distributional justice in solar energy implementation in India: the case of Charanka solar park. J Rural Stud.

